# Resistance to Lefamulin: An Evaluation of Data from In Vitro Antimicrobial Susceptibility Studies

**DOI:** 10.3390/antibiotics15010058

**Published:** 2026-01-05

**Authors:** Matthew E. Falagas, George Fanariotis, Laura T. Romanos, Konstantinos M. Katsikas, Stylianos A. Kakoullis

**Affiliations:** 1 Alfa Institute of Biomedical Sciences (AIBS), 151 23 Athens, Greece; g.fanariotis@aibs.gr (G.F.); l.romanos@aibs.gr (L.T.R.); k.katsikas@aibs.gr (K.M.K.); 2 Department of Medicine, School of Medicine, European University Cyprus, Nicosia 1516, Cyprus; s.kakoullis@euc.ac.cy; 3 Department of Medicine, School of Medicine, Tufts University, Boston 02111, MA, USA; 4 Department of Medicine, Hygeia Hospital, 151 23 Athens, Greece

**Keywords:** BC-3781, community-acquired bacterial pneumonia, *H. influenzae*, lefamulin, *S. aureus*, *S. pneumoniae*, pneumonia

## Abstract

Lefamulin, a new, first-in-class pleuromutilin antibiotic, was recently approved by the Food and Drug Administration (FDA) and European Medicines Agency (EMA) for the treatment of patients with community-acquired bacterial pneumonia (CABP). In this context, this review aimed to evaluate its activity against the most common pathogens causing this infection. A thorough search was performed in five databases (Embase, Scopus, Web of Science, PubMed, PubMed Central) from their inception to 14th of October 2025. Clinical and Laboratory Standards Institute (CLSI) and European Committee on Antimicrobial Susceptibility Testing (EUCAST) resistance breakpoints were applied. Out of a total of 224 articles identified, 11 were deemed eligible for inclusion. Resistance among *Streptococcus pneumoniae*, *Haemophilus influenzae*, and *Staphylococcus aureus* isolates was 0–2.6%, 0–2.4%, and 0–4.3%, respectively. Even among isolates with specific mechanisms of resistance, such as β-lactamase-producing *H. influenzae* and methicillin-resistant *S. aureus*, resistance was below 2.4% and 3.4%, respectively. Among isolates for which no breakpoints were available (*Moraxella catarrhalis,* atypical pathogens, *Enterococcus* spp., *Streptococcus* spp., *Haemophilus* spp., and *Staphylococcus* spp.), MIC_90_ values were low. An exception were isolates belonging to *Enterococcus* spp., which displayed MIC_90_ values ranging from 0.25 to >16 mg/L in the two studies with relevant data. Lefamulin demonstrated broad in vitro activity against key pathogens causing CABP, making it a considerable addition to the therapeutic options for such infections, especially in cases where first-line agents cannot be used for reasons such as allergy or previous failure.

## 1. Introduction

Pneumonia, a common infectious entity in clinical practice, remains a major contributing factor to the global morbidity and mortality [1]. The 2021 Global Disease Burden report states that pneumonia has caused over 2.1 million deaths in 2021, with populations at the extremes of age being at greater risk [2,3].

Community-acquired pneumonia (CAP), defined as the development of a lung infection outside the hospital setting or within ≤48 h of admission, is the more common variant of the disease and carries considerable clinical and economic implications [4]. Common risk factors for CAP include increasing age, chronic pulmonary comorbidities, and high levels of long-term care. Additionally, immunosuppression (such as that associated with solid organ transplantation, HIV infection, and high-dose systemic steroid therapy) is an important factor related to CAP, not only to its occurrence but also to mortality [5]. This syndrome results in millions of outpatient and emergency department visits and over 1 million hospitalizations each year in the US [6,7]. Despite most cases being managed in outpatient settings, a proportion of patients, with considerable variation among developed countries, requires hospitalization, with a short-term mortality ranging from 4% to 18% for hospitalized patients and reaching 50% for patients in intensive care units, and total CAP-related inpatient costs of more than 7.5 billion dollars annually [4,8,9,10].

The etiology of CAP cannot be identified in up to 62% of all patients who have radiographic evidence of pneumonia and specimens available for bacterial and viral testing. Even after using specific diagnostic methods such as culture, antigen detection, and molecular tests, the probability of pathogen detection is only 38%. The reason may be prior antibiotic use, the inability to obtain high-quality lower respiratory tract specimens, selection of insensitive or non-specific tests, or viral etiology [11].

However, it is well established that most cases are caused by viral and bacterial pathogens [12]. Community-acquired bacterial pneumonia (CABP) accounts for 30–50% of all cases and involves both typical and atypical bacteria [13,14]. There are several risk factors for CABP. *Streptococcus pneumoniae* remains the most frequent causative organism overall, but with a gradually decreasing incidence, probably due to the extending pneumococcal vaccination program [15]. *Haemophilus influenzae* and *Moraxella catarrhalis*, among the typical bacteria, along with atypical bacteria such as *Mycoplasma pneumoniae* and *Chlamydia pneumoniae*, constitute the remaining bacteria of the leading group of pathogens responsible for most CABP cases. Additionally, *Staphylococcus aureus,* including methicillin-resistant *S. aureus* (MRSA), is a prominent cause of CABP in specific clinical contexts. *S. aureus* CABP is more common following viral respiratory tract infections and among patients with risk factors for MRSA infection, such as prior MRSA lower respiratory tract infection or colonization and residence in close-contact facilities [11,16].

Given this broad etiological microbial spectrum of CABP, empiric antibiotic therapy, the mainstay of current treatment, should not only be tailored to regional epidemiologic trends but also consider risk factors for infection with antimicrobial-resistant organisms, local antimicrobial resistance (AMR) patterns, and disease severity [17]. Although the proportion of pneumococcal strains non-susceptible to β-lactams, macrolides, or fluoroquinolones varies significantly by region [18], their presence in the community complicates the selection of a suitable antibiotic regimen, as does the presence of *H. influenzae* and *M. catarrhalis* β-lactamase-producing strains [19]. In addition, the relatively uncommon community-associated MRSA pneumonia and the subsequent risk of developing severe outcomes, even in young healthy adults, warrant adequate coverage of these strains by empiric treatments in cases where infection by such strains of *Staphylococcus* spp. is suspected, including in patients with pneumonia after viral respiratory tract infections [20,21].

*S. pneumoniae* isolates have been displaying increasing resistance to macrolides such as azithromycin (even up to 40% non-susceptibility) [22]. They have also been shown to develop mechanisms of resistance to β-lactams and fluoroquinolones [23]. *Haemophilus* spp. and *M. catarrhalis* isolates may also be resistant to macrolides and quinolones [24,25]. Studies have shown that even atypical pathogens that cause CABP, such as *M. pneumoniae,* may be resistant to commonly used antibiotics such as macrolides [26]. Developments regarding the increasing resistance of CABP-causing pathogens to mainstay treatments highlight the need for the development of novel antibiotic agents.

Pleuromutilins are natural antimicrobial agents that were initially isolated from the fungus *Clitophilus scyphoides* in the 1950s [27]. Their molecules consist of a tricyclic core, a C21 ketone group, and various C14 side chains [28]. Naturally occurring alterations at the C14 position led to the development of early semisynthetic pleuromutilin derivatives, tiamulin and valnemulin, both approved for veterinary use only. Further structural modifications to the C14 chain led to the discovery of lefamulin (formerly known as BC-3781), which is the first semisynthetic pleuromutilin used for the systemic treatment of bacterial infections in humans [29].

Lefamulin inhibits bacterial protein synthesis by binding to the peptidyl transferase center (PTC) on the 50S ribosomal subunit through an ‘induced-fit mechanism’. The drug’s tricyclic core and the C14 side chain bind to the A (aminoacyl) and P (peptidyl) sites of the ribosome, respectively, via hydrophobic and hydrogen bonds. This binding disrupts the proper positioning of the tRNA, thereby inhibiting chain elongation, specifically during the formation of the first peptide bond. After chain elongation begins, lefamulin is no longer effective [28,30].

The C14 side chain plays a crucial role in the drug’s pharmacodynamic and antimicrobial properties [31]. An additional hydrogen bond forms between the NH_2_ group of the C14 extension and the A2062 nucleotide of the 23S rRNA. This improves the stability of the lefamulin–ribosome complex, thereby enhancing the drug’s inhibitory potency relative to other pleuromutilins [32].

Lefamulin, which has demonstrated in vitro activity against typical pathogens that cause CABP, was approved by the US Food and Drug Administration (FDA) (August 2019) and the European Medicines Agency (EMA) (July 2020) for the treatment of patients with CABP caused by susceptible organisms. It is available in an oral and intravenous formulation and is active against organisms such as *S. pneumoniae*, *S. aureus* (only for methicillin-susceptible isolates according to the FDA), *H. influenzae*, *Legionella pneumophila*, *M. pneumoniae*, and *C. pneumoniae*. It is also currently being evaluated for use in the treatment of other kinds of infections. These include osteomyelitis, healthcare-acquired bacterial pneumonia/ventilator-associated bacterial pneumonia, prosthetic joint infections, sexually transmitted infections, and acute bacterial skin and skin structure infections [27,33,34,35].

In this review, we aimed to evaluate lefamulin’s activity against common pathogens that cause CABP and to determine the proportion of pathogens resistant to it. Such information may help clinicians utilize it safely and effectively in clinical practice.

## 2. Methods

### 2.1. Sources and Search Strategy

**Figure 1:**
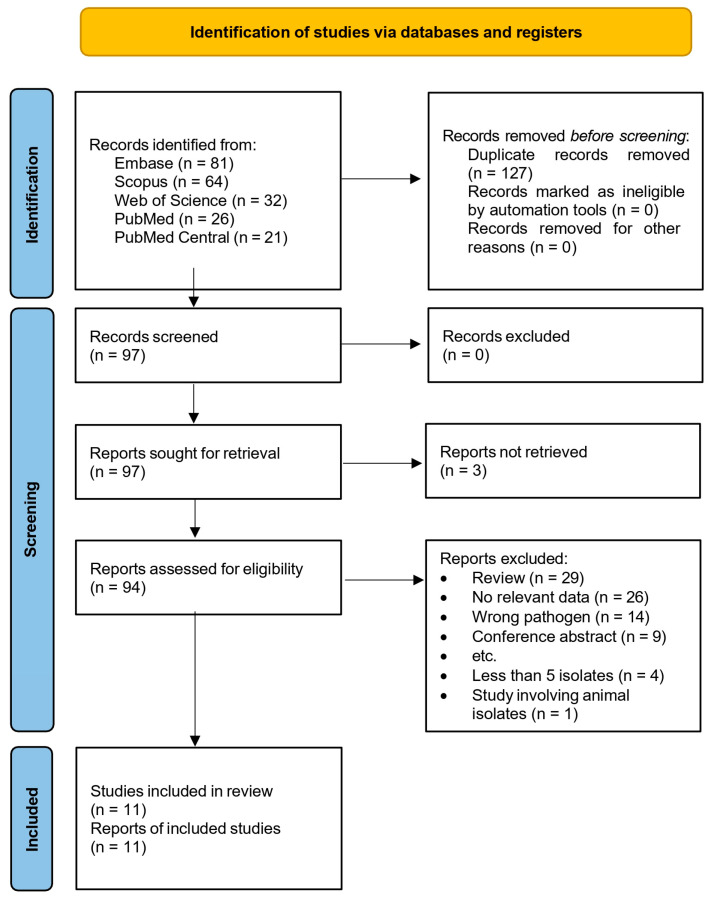
Flow diagram for the evaluation, selection, and inclusion of relevant articles.

The flow diagram for evaluating, selecting, and including relevant articles is shown in Figure 1. We did not upload the study protocol to a relevant registry. An extensive literature review was performed across five databases (Embase, Scopus, Web of Science, PubMed, PubMed Central) from their inception to the 14th of October 2025. In Supplementary File S1, the detailed search strategy is presented. Terms such as “lefamulin”, “resistance”, “non-susceptibility”, “MIC”, and “disk diffusion” were used.

### 2.2. Eligibility Criteria

Two inclusion criteria were defined. More specifically, they were as follows: (a) the terms “lefamulin” or “BC-3781” were included in the title/abstract/keywords, and (b) the study reported minimum inhibitory concentration (MIC) or disk diffusion susceptibility data. Six exclusion criteria were applied: (a) non-primary research articles; (b) articles using isolates from animal sources; (c) case reports of a single isolate or patient; (d) conference abstracts; (e) primary research articles that did not contain relevant data for this review; (f) studies evaluating ≤5 total isolates, for lefamulin susceptibility testing.

### 2.3. Screening of Studies

A total of 224 articles were identified; after deduplication, 97 remained for screening. We utilized the Rayyan tool to perform deduplication based on digital object identifiers (DOIs). Subsequently, two reviewers (L.T.R. and K.M.K.) independently screened all retrieved records by reviewing their full texts.

### 2.4. Data Extraction and Tabulation

Three independent reviewers extracted the necessary data and tabulated it (L.T.R., G.F., K.M.K.). We included data on the total number of isolates studied, the number of isolates per species, the MIC range, MIC_50_, and MIC_90_. We also included the number of isolates and the proportion of all isolates that were resistant to lefamulin. Whenever percentages were provided as the only measure of lefamulin resistance in a study, we calculated the corresponding number of resistant isolates based on the total number of isolates and the given percentage, and vice versa. In instances where authors did not provide resistance data, the number and proportion of resistant isolates were calculated using the published resistance breakpoints, where feasible. MIC_50_ and MIC_90_ were also calculated whenever possible, in cases where that data was not presented in the included articles. Data were grouped by bacterial species (*S. pneumoniae*, *H. influenzae*, *S. aureus*, and isolates belonging to other species). In addition, we extracted data on the proportions of resistance, or proportions of non-susceptibility of CABP-associated pathogens to common antibiotics (along with data for lefamulin), currently used for the empiric treatment of CABP. Any disagreements between reviewers were resolved by consensus with a senior author (M.E.F.).

### 2.5. Breakpoints of Susceptibility Testing

The European Committee on Antimicrobial Susceptibility Testing (EUCAST) and the Clinical and Laboratory Standards Institute (CLSI) breakpoints of resistance were utilized. According to both organisms, the breakpoint of resistance for *S. pneumoniae* and *S. aureus* is MIC > 0.5 mg/L and MIC > 0.25 mg/L, respectively. CLSI has also published a breakpoint of resistance of *H. influenzae*, which is MIC > 2 mg/L. We utilized the EUCAST Breakpoint tables for interpretation of MICs and zone diameters (Version 15.0) and the 2024 version of the CLSI M100 Performance Standards for Antimicrobial Susceptibility Testing.

## 3. Results

### 3.1. Selection of Relevant Articles

After the exclusion of 83 articles that did not fulfill the inclusion criteria and three that were not retrieved, 11 remained eligible [36,37,38,39,40,41,42,43,44,45,46].

### 3.2. Main Findings

In Table 1, we present data on the resistance of *S. pneumoniae* isolates to lefamulin [36,38,40,41,42,43,45,46]. Overall, resistance among all *S. pneumoniae* isolates ranged from 0% to 2.6% [36,38,40,41,42,43,45,46]. Specifically, penicillin-susceptible and intermediate isolates (usually defined as MIC ≤ 0.06 mg/L and MIC ≤ 0.12–1 mg/L by the authors of the included studies, respectively) showed resistance to lefamulin ranging from 0% to 0.2% [36,38,40,41,42,46]. Penicillin-resistant isolates (usually defined as isolates with MIC ≥ 2 mg/L by the authors) showed 0% resistance to lefamulin [36,38,40,41,42,46]. Macrolide-resistant isolates (defined as erythromycin MIC ≥ 1 mg/L) showed 2.6% resistance in one study of 3844 isolates [42].

Data on the antimicrobial activity of lefamulin against multidrug-resistant (MDR) and extensively drug-resistant (XDR) isolates were also available in two studies [42,43]. MDR and XDR isolates in these studies were defined as non-susceptible isolates to ≥3 and ≥5 classes of the following antimicrobial agents, respectively: penicillin (MIC ≥ 4 mg/L), ceftriaxone (MIC ≥ 2 mg/L), erythromycin (MIC ≥ 0.5 mg/L), clindamycin (MIC ≥ 0.5 mg/L), levofloxacin (MIC ≥ 4 mg/L), tetracycline (MIC ≥ 2 mg/L), and trimethoprim-sulfamethoxazole (MIC ≥ 1 mg/L). XDR isolates showed a resistance of 0% while MDR isolates showed 0.2% in both studies, with relevant data [42,43,47].

In Table 2, we present data on the resistance of *H. influenzae* isolates to lefamulin [36,38,40,41,42,43,46]. Overall, *H. influenzae* isolates showed resistance ranging from 0% to 2.4% [36,38,40,41,42,43,46]. Among β-lactamase *H. influenzae* negative isolates, resistance to lefamulin ranged from 0% to 1.5% [36,38,40,41,43,46], while among β-lactamase-positive isolates it ranged from 0% to 2.4% [36,38,40,41,42,43,46].

In Table 3, we present data on the resistance of *S. aureus* isolates to lefamulin [36,38,39,41,42,43,46]. Overall, *S. aureus* isolates showed resistance ranging from 0% to 4.3% [36,38,39,41,42,43,46]. Specifically, MSSA and MRSA isolates showed resistance of 0–4.3% and 0–3.4%, respectively [36,38,39,41,42,43,46]. MDR *S. aureus isolates* (defined as methicillin-, azithromycin-, and levofloxacin-resistant isolates) showed a resistance to lefamulin of 0.2% among the 610 studied isolates [42].

In Table 4, Table 5 and Table 6, we present data on isolates of species for which the CLSI or EUCAST has defined no resistance breakpoints. More specifically, Table 4 contains data on *M. catarrhalis* isolates [36,38,40,41,42,46]. In one study, the authors used the resistance breakpoint of MIC ≤ 0.5 mg/L (the susceptibility criteria for lefamulin were determined based on the CLSI M45 guideline) for 81 *M. catarrhalis* isolates and reported 0% resistance of these isolates to lefamulin [46].

Table 5 presents data on isolates belonging to *Streptococcus* spp., except for *S. pneumoniae*, which was presented separately in Table 1 [36,39,41,42,44].

Table 6 contains data from the remaining isolates studied (belonging to atypical pathogens as well as *Enterococcus* spp., *Haemophilus* spp., and *Staphylococcus* spp.). (except for data about *H. influenzae* and *S. aureus,* which were presented separately in Table 2 and Table 3, respectively) [36,37,39,40,41,42,46].

In Table 7, we present the proportions of resistance, or proportions of non-susceptibility of CABP-causing pathogens to selected antibiotics as well as to lefamulin. The selected antimicrobial agents represent three of the most used antibiotic classes for the empiric treatment of CABP, specifically, β-lactams, macrolides, and fluoroquinolones [1]. The data shown in the table allow for a comparison between the in vitro activity of these agents. The resistance proportions to lefamulin are less than 0.4% for *S. pneumoniae* isolates among all studies [36,38,41,42,43,45,46]. For the same isolates, the corresponding proportions of resistance to ceftriaxone and fluoroquinolones are within the lower end of the single-digit range, but still higher than the resistance proportions to lefamulin [36,38,41,42,43,45,46]. Higher resistance proportions of *S. pneumoniae* isolates, often more than 10%, are noted with penicillin and amoxicillin-clavulanate, while considerably higher resistance proportions are recorded with azithromycin, ranging from 33.6% to 94.5% across all studies [36,38,41,42,43,45,46].

The presented data on the comparative activity of the widely used antimicrobials against lefamulin reveal a similar trend among other CABP-causing pathogens, beyond *S. pneumoniae*, such as *H. influenzae* and *S. aureus*. In particular, this pattern becomes more evident in the subset of MRSA isolates, where lefamulin resistance proportions are in the single digits, while the resistance to other antibiotic classes is considerably high, even in the case of the fluoroquinolones [36,38,41,42,43,46].

## 4. Discussion

Overall, the percentage of resistance of all studied isolates for which resistance breakpoints have been defined was less than 4.6%. Even among the rest of the isolates, MIC values were mostly low. Specifically, among *M. catarrhalis* isolates, all MIC_90_ values were ≤0.5 mg/L. Among the non-*S. pneumoniae Streptococcus* spp. isolates, all MIC_90_ values were ≤2 mg/L. Regarding atypical bacteria, MIC_90_ values were ≤0.5 mg/L, and among *Staphylococcus* spp. (except *S. aureus*) isolates, they were even lower, with the maximum MIC_90_ being 0.25 mg/L. In the two studies that included *Haemophilus parainfluenzae* isolates, MIC_90_ were 4 and 1 mg/L, respectively. Lastly, among *Enterococcus* spp. isolates, which were analyzed in two studies, MIC_90_ values ranged from 0.25 to >16 mg/L, suggesting that this drug may not be as effective against such pathogens, especially those that have acquired antimicrobial resistance mechanisms.

The data on the comparative in vitro antimicrobial activity of lefamulin against widely used antimicrobial agents for the empiric treatment of CABP confirmed the superior in vitro susceptibility of common CABP-causing bacteria to lefamulin, including *S. pneumoniae* and *H. influenzae*. A thorough analysis of the presented percentages reveals that the proportions of resistance to the other commonly used antibiotics were higher than those to lefamulin in all cases [36,38,40,41,42,43,45,46].

Pleuromutilins selectively inhibit prokaryotic protein synthesis with high specificity, without impacting eukaryotic protein translation [29]. This selective inhibition accounts for the particularly low incidence of cross-resistance to other major antibiotic classes, including protein synthesis inhibitors such as tetracyclines, macrolides, ketolides, fusidic acid, oxazolidinones, and lincosamides [31].

Lefamulin’s unique mechanism of action, combined with a very low spontaneous mutation frequency (≤10^−9^), indicates a low chance of resistance developing during clinical treatment [30]. However, resistance to lefamulin has been observed in vitro, mainly due to alterations in the ribosomal target site, which develop slowly and in a stepwise manner, requiring multiple mutations to reach high-level resistance. Mutations in 23S rRNA, as well as in the rplC and rplD genes, which encode ribosomal proteins L3 and L4, respectively, have been identified as the primary mechanism of resistance to lefamulin.

Mutations in the 23S RNA gene (rrn) confer resistance to lefamulin in isolates with a single copy of 23S rRNA, such as *Mycoplasma* spp. and *Brachyspira* spp. [52]. A single mutation in the 23S rRNA is sufficient for *Mycoplasma* spp. to develop high-level resistance to lefamulin. Conversely, staphylococcal and streptococcal species with multiple copies of 23S rRNA are unlikely to develop significant resistance through a single mutation [30,52]. Additionally, mutations and deletions in the rplC and rplD genes also contribute to lefamulin resistance. Although L3 and L4 proteins do not directly interact with the drug, changes in these proteins can cause structural alterations in the PTC. As a result, the proper positioning of lefamulin is disrupted, leading to decreased binding efficiency [52].

Efforts to reduce the risk of developing resistance to this antibiotic include adherence to basic principles of antimicrobial chemotherapy. The appropriate dosage and duration of antimicrobial treatment ensure the highest possible probability of clinical success and microbiological eradication, as well as the lowest possible probability of mutagenicity and the development of antimicrobial resistance.

Two multinational, multicenter clinical trials have illuminated the safety and efficacy profile of lefamulin for the treatment of CABP [53]. The LEAP-1, a 2015 Phase 3 clinical trial (NCT02559310), compared lefamulin to moxifloxacin, with or without linezolid, in a study population of 551 persons. Moxifloxacin was chosen as a comparator agent, as it is an extended-spectrum fluoroquinolone with activity that includes Gram-positive cocci and atypical bacteria, which commonly cause CABP. It is one of the recommended fluoroquinolones for the treatment of this infection. Early clinical response (ECR) and investigator’s assessment of clinical response (IACR) were the primary and secondary endpoints, respectively. The definition of ECR was survival with improvement in at least two signs and symptoms of CABP, no worsening of any CABP sign or symptom, and no use of concomitant antibiotics for the treatment of CABP through the ECR assessment. This outcome was assessed 96 ± 24 h after the first dose of study drug. Patients in the lefamulin group received 150 mg intravenously (IV) every 12 h, while participants in the comparator group received moxifloxacin 400 mg IV once daily. After 3 days of treatment, the switch to the equivalent oral formulation of the intravenous agents was introduced if predefined criteria were met. When MRSA was suspected during screening, linezolid was added to moxifloxacin (but not in the lefamulin group) [54].

The proportion of patients achieving ECR did not differ significantly between treatment arms (87.3% with lefamulin and 90.2% with moxifloxacin). Regarding the secondary outcome, in the intention-to-treat population, 81.7% of patients treated with lefamulin and 84.2% treated with moxifloxacin showed adequate clinical improvement without requiring additional antibacterial therapy [54]. Furthermore, treatment-emergent serious adverse events occurred in 7.0% of patients receiving lefamulin and in 4.8% of patients receiving moxifloxacin. Non-serious adverse events developed in 14.3% of participants treated with lefamulin and in 16.9% of patients treated with moxifloxacin. The all-cause mortality was 1.8% and 2.2% in the lefamulin and the moxifloxacin arm, respectively (five and six deaths, respectively). None of these deaths was deemed to be related to the study drug [54,55].

The LEAP-2 clinical trial (NCT02813694) evaluated the safety and efficacy of lefamulin versus moxifloxacin in 738 adults with moderate community-acquired pneumonia. It used the same primary and secondary outcomes as the LEAP 1 trial. This double-blind, randomized trial compared 600 mg of oral lefamulin with 400 mg of oral moxifloxacin, both administered once daily. The same percentage (90.8%) of participants in both arms demonstrated ECR. Also, no statistically significant difference was noted between the two arms in the secondary outcome either; 87.5% and 89.1% of participants in the lefamulin and the moxifloxacin group, respectively, showed improvement or resolution of clinical symptoms and signs, such that no additional antibacterial therapy was administered for the treatment of the current CABP episode. In addition, treatment-emergent adverse events were reported in 32.6% of patients in the lefamulin group and 25% of patients in the moxifloxacin group, most of which were mild or moderate in severity. The incidence of serious adverse events was 4.6% with lefamulin and 4.9% with moxifloxacin, while all-cause mortality was 1.4% and 0.8% in the lefamulin and moxifloxacin groups, respectively [56,57].

These clinical trials established the non-inferiority of lefamulin compared with moxifloxacin for the treatment of CABP, and they provided insight into the types and frequencies of treatment-emergent and treatment-related adverse effects. Adverse events with an incidence of ≥2% were considered common by the investigators. The analysis of the pooled safety population revealed that overall, the proportion of patients with treatment-emergent adverse events was similar with lefamulin (34.9%) and moxifloxacin (30.4%). Most events were mild to moderate, and serious treatment-emergent adverse events developed in approximately 5% of participants in each group (5.6% and 4.8% in the lefamulin and moxifloxacin groups, respectively). The percentage of treatment-emergent adverse events, considered related to the study drug by the investigators, was 15.4% and 10.6% in the lefamulin and moxifloxacin groups, respectively. Treatment-emergent adverse events that led to study discontinuation occurred in 3.1% with lefamulin and 3.3% with moxifloxacin. The 28-day all-cause mortality was 1.2% and 1.1% in the lefamulin and the moxifloxacin group, respectively. None of these deaths was attributed to a treatment-related adverse event [53,54,56].

The most common adverse events, by system, were gastrointestinal (GI). (13.1% and 10.1% in the lefamulin and moxifloxacin groups, respectively). The GI adverse events associated with lefamulin compared to moxifloxacin included diarrhea (7.3% vs. 3.9%), nausea (4.2% vs. 2.0%), and vomiting (2.3% vs. 0.6%) [53]. In addition, a few other common adverse events were reported in the LEAP-1 trial, which, as previously noted, evaluated the safety of an initial 3-day IV lefamulin course followed by transition to oral therapy. These adverse events of lefamulin compared to moxifloxacin included administration-site reactions (7% vs. 3%), hepatic enzyme elevation (3% vs. 3%), hypokalemia (3% vs. 2%), and insomnia (3% vs. 2%) [54,55].

Cardiac treatment-emergent adverse events occurred in 2.5% and 3.1%, in the lefamulin and moxifloxacin groups, respectively. Mean QT intervals corrected by Fridericia’s method increased from baseline in both treatment groups. However, the mean [SD] maximum change from baseline was numerically lower for lefamulin (16.9 [16.9] ms) compared with moxifloxacin (19.3 [17.7] ms). No arrhythmias associated with QT prolongation were reported in these trials [53].

In addition to the common adverse events, other events occurring in fewer than 2% of patients treated with lefamulin were reported. These included anemia, thrombocytopenia, palpitations, atrial fibrillation, *Clostridium difficile* colitis, oropharyngeal candidiasis, vulvovaginal candidiasis, increased creatine phosphokinase, increased gamma-glutamyl transferase, somnolence, anxiety, and urinary retention [55,57].

The results of these two Phase 3 non-inferiority trials served as the basis for lefamulin’s FDA approval for the treatment of adults with CABP due to the following pathogens: *S. pneumoniae*, *S. aureus* (methicillin-susceptible isolates), *H. influenzae*, *L. pneumophila*, *M. pneumoniae*, and *C. pneumoniae* [58]. This list does not include *M. catarrhalis*, a common cause of CABP, and MRSA, which, albeit less common, is especially clinically relevant in post-viral CABP. In the LEAP-1 and LEAP-2 studies, a baseline pathogen was identified in 55% (709/1289) of enrolled patients. Yet, *M. catarrhalis* was isolated in 68 cases (9.5%) overall, and among these patients, 46 received lefamulin. On the other hand, *S. aureus* was identified in only three cases [55,57]. Given these data, the FDA approval label for lefamulin lists both pathogens under the subsection for which the efficacy in clinical infections has not yet been established in adequate and well-controlled clinical trials, even though they have exhibited substantial in vitro activity [34]. Similarly, the EMA approval label reports *M. catarrhalis* under the section of bacteria that are probably susceptible to lefamulin based on in vitro studies, without proven clinical efficacy [35].

Current therapeutic options for CABP include β-lactam, tetracycline, macrolide, and quinolone antibiotics. Previous studies have investigated the comparative effectiveness and safety of antibiotics of these classes, in monotherapy and in combination antimicrobial therapy. The high frequency of atypical bacteria as etiologic agents of CABP suggests that antibiotics active against these pathogens, especially macrolides, should be considered. Meta-analyses and guidelines have provided clinicians with data on the appropriate use of antibiotics for the treatment of patients with CABP [59,60,61,62,63,64,65]. Relevant data from these resources indicate that quinolones with activity against respiratory tract pathogens should be reserved for patients with severe CABP or significant comorbidities. In this context, lefamulin, when available, offers an additional therapeutic option for patients with CABP.

Epidemiologic data on resistance trends among the most common CABP-causing bacteria can assist clinicians in determining the role of lefamulin in the current treatment landscape. In the US, it is estimated that 20–40% of *S. pneumoniae* isolates demonstrate resistance to macrolides [66]. Similarly, non-susceptibility to β-lactams is increasing and represents a well-recognized challenge in the treatment of CABP. However, the proportion of *S. pneumoniae* isolates resistant to β-lactams shows considerable variability across classes within this broad family of antimicrobials [23]. Notably, up to 40% of *S. pneumoniae* isolates can be resistant to penicillin, while the corresponding proportion for isolates resistant to cephalosporins ranges from less than 1%, in the case of ceftaroline, to approximately 30%, in the case of cefuroxime [67]. Fortunately, fluoroquinolones, a major class of antibiotics considered for empiric treatment of CABP in patients with specific characteristics (severe CABP or significant comorbidities), have favorable susceptibility profiles, with resistance ranging from less than 1% to 2% [67].

Additional systematic reviews and meta-analyses, along with data from large antimicrobial surveillance studies, provide valuable insight into the epidemiologic trends of other common CABP-causing pathogens and their susceptibility to currently available antibiotics. According to a recent meta-analysis, the prevalence of *H. influenzae* resistance to macrolides can reach up to 12.6% with clarithromycin, and 9.3% with azithromycin [68]. Furthermore, data from the SENTRY antimicrobial surveillance program have shown that 28.3% of *H. influenzae* isolates were resistant to ampicillin and 8.6% of β-lactamase-positive isolates were resistant to amoxicillin-clavulanate [19]. Regarding cephalosporins, notable resistance to cefuroxime has been observed, with a reported proportion up to 19.1% in MDR *H. influenzae* isolates [69]. Some studies from centers in China have reported similar or even higher resistance among *H. influenzae* isolates, specifically, 19.8% among isolates collected from adult patients with CABP and over 50% in a pediatric population of approximately 21,700 patients [70,71]. Also, high resistance to trimethoprim-sulfamethoxazole has been observed, with an estimated proportion up to 65.2% [19]. On the contrary, the non-susceptibility of *H. influenzae* to fluoroquinolones is less than 1% with levofloxacin, and less than 2% with moxifloxacin [19].

In addition to the aforementioned causative organisms, the therapeutic spectrum of lefamulin extends to other CABP-causing pathogens, such as the atypical bacteria *M. pneumoniae* and *C. pneumoniae*. Macrolides have been traditionally the drug-of-choice for the treatment of CABP associated with *M. pneumoniae*. However, *M. pneumoniae* resistance to macrolides has been well documented, with an estimated proportion ranging from 2.1% to 18.3% in the US, and from 35% to 67% in Asia over the last 15 years [26,72]. Antimicrobials, such as tetracyclines and respiratory fluoroquinolones, provide reliable alternatives to macrolides, as almost all clinical isolates remain susceptible to both classes of antibiotics [73]. *C. pneumoniae*, an obligate intracellular atypical bacterium, can be treated successfully with antimicrobials, achieving adequate intracellular penetration [74]. The standard antibiotics for *C. pneumoniae*-associated pneumonia include macrolides, tetracyclines, and fluoroquinolones. These antibiotics have preserved excellent efficacy against clinical isolates through the years and remain the preferred antibacterials for respiratory infections caused by *C. pneumoniae* [75]. *L. pneumophila* is intrinsically resistant to most β-lactam antibiotics due to β-lactamase production and poor intracellular penetration. However, it is susceptible to macrolides and fluoroquinolones, with evidence of emerging resistance to these antibiotics being rare and clinically insignificant [76,77]. Lastly, MSSA isolates, clinically relevant in post-viral CABP, are non-susceptible to penicillin G and aminopenicillins, with global resistance of *S. aureus* isolates to these antibiotics exceeding 80% [78]. Nevertheless, antistaphylococcal penicillins and first-generation cephalosporins retain excellent efficacy against MSSA, whereas resistance to erythromycin, clindamycin, and ciprofloxacin ranges from 20% to 25%, 7% to 10%, and 5% to 10%, respectively [79,80].

Supplementary to these epidemiologic data, the resistance percentages summarized in Table 7 can further assist clinicians in their decision-making. The listed resistance percentages are in accordance with the aforementioned epidemiologic trends and demonstrate the alarmingly high resistance of *S. pneumoniae* isolates to penicillins and macrolides. The non-susceptibility proportions of *S. pneumoniae* to penicillin and azithromycin range from 18.6% to 79.8% and 33.6% to 94.5%, respectively [36,38,40,41,42,43,45,46]. The fluoroquinolones and ceftriaxone, a major 3rd-generation cephalosporin, retain considerable activity against *S. pneumoniae* isolates but are still inferior to lefamulin [36,38,40,41,42,43,45,46]. A similar resistance pattern, favoring the use of lefamulin, is identified among *H. influenzae* isolates, although the resistance of *H. influenzae* isolates to azithromycin is not as widespread as the corresponding resistance of *S. pneumoniae* to azithromycin [36,40,41,42,43,45,46]. *S. aureus* isolates, particularly the subset of MRSA isolates, have emerged as a growing problem for which even the highly active fluoroquinolone class of antibiotics demonstrates inadequate activity [36,38,40,41,42,43,45,46]. Fortunately, lefamulin shows low resistance proportions against these isolates, too [36,38,41,42,43,46].

Our article is not without limitations. We did not register our study protocol in a relevant database. We also did not perform a risk of bias assessment as no available tool is available for in vitro susceptibility studies. Additionally, in several studies, we extracted the number and, subsequently, the proportion of resistant isolates from the reported MIC distributions of the analyzed isolates, using the published CLSI and EUCAST breakpoints. Lastly, two studies partially overlapped, as they both used isolates from the “SENTRY” antimicrobial surveillance program; however, because they evaluated largely different subsets of isolates, they were included in our review [42,43].

## 5. Conclusions

This analysis of published in vitro susceptibility studies of clinical isolates to lefamulin reveals limited resistance to *S. pneumoniae*, *H. influenzae*, and methicillin-susceptible *S. aureus* isolates. Additionally, considerable in vitro activity has been demonstrated against *L. pneumophila* and atypical bacteria. Likewise, the considerable susceptibility of other CABP-causing pathogens, including *M. catarrhalis* and MRSA, as evidenced by the low reported MIC values and ranges, encourages further research through clinical trials to elucidate the therapeutic role of lefamulin against these pathogens. For now, clinicians can consider lefamulin an option for the initial treatment of adults with CABP due to specific pathogens, given its demonstrated in vitro activity and promising trial results. It could be especially beneficial for patients who either have had a previous adverse reaction or allergy to the agents usually chosen for such infections, such as β-lactams, macrolides, and quinolones. Additionally, it could be used in cases where these agents have already been used and have been ineffective.

## Figures and Tables

**Table 1:** Resistance of *S. pneumoniae* isolates to lefamulin.

Author *	Year	Isolates	N	MIC Range/Value (mg/L)	MIC_50_ (mg/L)	MIC_90_ (mg/L)	Resistance *n* (% **)
Ding [46]	2025	*S. pneumoniae*	529	≤0.015–0.25	0.125	0.125	0 (0)
		penicillin-R	287	≤0.015–0.25	0.125	0.125	0 (0)
		penicillin-S	134	≤0.015–0.25	0.06	0.125	0 (0)
		penicillin-I	108	≤0.015–0.25	0.125	0.25	0 (0)
Paukner [42]	2023	*S. pneumoniae*	10,586	0.008–2	0.06	0.25	10 (0.09)
		penicillin-S ^a, b^	6775	0.008–2	0.06	0.25	6 (0.08)
		macrolide-R ^c^	3844	0.008–2	0.12	0.25	10 (2.6)
		MDR ^d^	2342	0.008–1	0.06	0.12	5 (0.2)
		penicillin-I ^a, e^	2292	0.008–1	0.12	0.25	4 (0.2)
		penicillin-R ^a, f^	1519	0.008–0.5	0.06	0.12	0 (0)
		XDR	553	0.015–0.25	0.06	0.12	0 (0)
Taylor [38]	2021	*S. pneumoniae*	482	≤0.004–0.25	0.12	0.12	0 (0)
		penicillin-S	397	≤0.004–0.25	0.12	0.12	0 (0)
		clarithromycin-R	110	0.008–0.25	0.12	0.25	0 (0)
		doxycycline-R	67	0.015–0.25	0.12	0.25	0 (0)
		penicillin-I	64	0.008–0.25	0.12	0.25	0 (0)
		penicillin-R	21	0.06–0.25	0.12	0.12	0 (0)
Wu [36]	2020	*S. pneumoniae* ^g^	172	≤0.015–0.25	≤0.015	≤0.25	0 (0)
		penicillin-R ^f^	118	≤0.015–0.25	≤0.015	≤0.015	0 (0)
		penicillin-I ^e^	40	0.03–0.25	0.125	0.25	0 (0)
		penicillin-S ^b^	40	≤0.015	≤0.015	≤0.015	0 (0)
Paukner [43]	2019	*S. pneumoniae*	3923	≤0.008–1	0.06	0.12	5 (0.1)
		MDR ^d^	821	≤0.008–1	0.06	0.12	2 (0.2)
		penicillin-NS ^h^	189	0.015–0.25	0.06	0.12	0 (0)
		XDR ^d^	181	0.015–0.25	0.06	0.12	0 (0)
		ceftriaxone-NS ^i^	155	0.015–0.25	0.06	0.12	0 (0)
		levofloxacin-NS ^h^	47	0.015–1	0.06	0.25	1 (2.1)
Mendes [45]	2016	*S. pneumoniae*	822	≤0.015–1	0.12	0.25	3 (0.4)
Paukner [41]	2013	*S. pneumoniae*	1473	≤0.008–1	0.12	0.25	3 (0.2)
		penicillin-S ^b^	903	≤0.008–1	NA	NA	2 (0.2)
		penicillin-R ^f^	312	0.015–0.5	NA	NA	0 (0)
		penicillin-I ^e^	258	0.015–1	NA	NA	1 (0.2)
Sader [40]	2011	*S. pneumoniae*	157	0.015–0.5	0.12	0.25	0 (0)
		penicillin-S ^b^	54	0.015–0.25	0.12	0.25	0 (0)
		penicillin-R ^f^	52	0.015–0.5	0.12	0.25	0 (0)
		penicillin-I ^e^	51	0.015–0.25	0.06	0.12	0 (0)

* Studies are presented in descending chronological order (and alphabetical order within a year). ** All percentage figures were rounded to one decimal place, except the values <0.1 that were rounded to two decimal places. Notes: ^a^ oral, non-meningitis, ^b^ MIC ≤ 0.06 mg/L; ^c^ erythromycin MIC ≥ 1 mg/L; ^d^ MDR and XDR status was based on nonsusceptibility to ≥3 and ≥5 classes, respectively, of the following antimicrobial agents, as described by Golden et al. and applying the following breakpoints: penicillin (MIC ≥ 4 mg/L), ceftriaxone (MIC ≥ 2 mg/L), erythromycin (MIC ≥ 0.5 mg/L), clindamycin (MIC ≥ 0.5 mg/L), levofloxacin (MIC ≥ 4 mg/L), tetracycline (MIC ≥ 2 mg/L), and trimethoprim-sulfamethoxazole (MIC ≥ 1 mg/L); ^e^ MIC ≤ 0.12–1 mg/L; ^f^ MIC ≥ 2 mg/L; ^g^ the reported sum of subsets of studied *S. pneumoniae* isolates based on the penicillin MIC is different than the reported total number of clinical *S. pneumoniae* isolates; ^h^ MIC > 4 mg/L; ^i^ MIC > 2 mg/L. Abbreviations: I, intermediate; MDR, multi-drug resistant; mg/L, milligram/liter; MIC, minimum inhibitory concentration; MIC_50_, MIC for inhibiting 50% of the isolates; MIC_90_, MIC for inhibiting 90% of the isolates; N, number of isolates tested; *n*, number of resistant isolates; NA, not available; NS, non-susceptible; R, resistant; S, susceptible; *S. pneumoniae*, *Streptococcus pneumoniae*; XDR, extensively drug resistant.

**Table 2:** Resistance of *H. influenzae* isolates to lefamulin.

Author *	Year	Isolates	N	MIC Range/Value (mg/L)	MIC_50_ (mg/L)	MIC_90_ (mg/L)	Resistance *n* (% **)
Ding [46]	2025	*H. influenzae*	121	≤0.015–2	0.5	1	0 (0)
		β-lactamase positive	63	0.25–2	0.5	1	0 (0)
		β-lactamase negative	58	≤0.015–2	0.5	1	0 (0)
Paukner [42]	2023	*H. influenzae*	3496	0.12–≥8	0.5	2	30 (0.9)
		β-lactamase positive	816	0.008–≥8	0.5	2	11 (1.3)
Taylor [38]	2021	*H. influenzae*	99	≤0.015–8	0.5	2	1 (1)
		β-lactamase positive ^a^	69	≤0.015–>8	0.5	2	1 (1.4)
		β-lactamase negative	30	≤0.015–2	0.5	2	0 (0)
Wu [36]	2020	*H. influenzae*	96	0.125–2	≤1	≤1	0 (0)
		β-lactamase negative	48	0.25–2	1	1	0 (0)
		β-lactamase positive	48	0.125–2	0.5	1	0 (0)
Paukner [43]	2019	*H. influenzae*	1086	≤0.12–8	0.5	1	10 (0.9)
		β-lactamase negative	835	≤0.12–4	0.5	1	9 (1.1)
		β-lactamase positive	251	≤0.12–8	0.5	1	1 (0.4)
Paukner [41]	2013	*H. influenzae*	360	0.015–8	1	2	6 (1.7)
		β-lactamase negative	275	0.15–8	NA	NA	4 (1.5)
		β-lactamase positive	85	0.25–8	NA	NA	2 (2.4)
Sader [40]	2011	*H. influenzae*	102	0.25–2	0.5	2	0 (0)
		β-lactamase negative	51	0.25–2	0.5	1	0 (0)
		β-lactamase positive	51	0.25–2	0.5	2	0 (0)

* Studies are presented in descending chronological order (and alphabetical order within a year). ** All percentage figures were rounded to one decimal place, except the values <0.1 that were rounded to two decimal places. Notes: ^a^ β-lactamase production for *H. influenzae* was analyzed according to the 2016 Clinical Microbiology Procedures Handbook. Abbreviations: *H. influenzae*, *Haemophilus influenzae*; N, number of Isolates; *n*, number of resistant isolates; NA, not available; MIC, minimum inhibitory concentration; MIC_50_, MIC for inhibiting 50% of the isolates; MIC_90_, MIC for inhibiting 90% of the isolates; mg/L, milligram/liter.

**Table 3:** Resistance of *S. aureus* isolates to lefamulin.

Author *	Year	Isolates	N	MIC Range/Value (mg/L)	MIC_50_ (mg/L)	MIC_90_ (mg/L)	Resistance *n* (% **)
Ding [46]	2025	*S. aureus*	306	≤0.015–>32	0.06	0.125	7 (2.3) ^a^
Paukner [42]	2024	*S. aureus*	3056	0.03–≥8	0.06	0.12	13 (0.4)
Paukner [43]	2019	*S. aureus*	2919	≤0.008–>32	0.06	0.12	11 (0.4)
Paukner [41]	2013	*S. aureus*	5527	0.015–>16	0.12	0.12	28 (0.5)
Sader [39]	2012	*S. aureus*	784	0.03–0.5	0.12	0.25	8 (1)
Ding [46]	2025	MSSA	188	≤0.015->32	0.06	0.125	3 (1.6) ^a^
Paukner [42]	2023	MSSA	2069	0.03–≥8	0.06	0.12	7 (0.3)
Taylor [38]	2021	MSSA	70	0.06–>2	0.12	0.25	3 (4.3)
Wu [36]	2020	MSSA	61	≤0.015–0.125	0.06	0.06	0 (0)
Paukner [43]	2019	MSSA	1981	≤0.03–>8	0.06	0.06	6 (0.3)
Paukner [41]	2013	MSSA	3157	0.015–>16	NA	NA	9 (0.3)
Sader [39]	2012	MSSA	253	0.03–0.5	0.12	0.12	1 (0.4)
Ding [46]	2025	MRSA	118	≤0.015–>32	0.125	0.125	4 (3.4) ^a^
Paukner [42]	2023	MRSA	987	0.03–≥8	0.06	0.12	6 (0.6)
Taylor [38]	2021	MRSA	130	0.06–>2	0.12	0.25	NA
Wu [36]	2020	MRSA	60	≤0.015−0.25	≤0.015	0.125	NA
Paukner [43]	2019	MRSA	938	≤0.03–>32	0.06	0.12	5 (0.6)
Paukner [41]	2013	MRSA	2370	0.015–4	0.12	0.25	19 (0.8)
Sader [39]	2012	MRSA	531	0.03–0.5	0.12	0.25	7 (1.3)
Paukner [42]	2023	MDR (methicillin-, azithromycin- and levofloxacin-R)	610	0.03–≥8	0.06	0.12	1 (0.2)

* Studies are presented in descending chronological order (and alphabetical order within a year). ** All percentage figures were rounded to one decimal place, except the values <0.1 that were rounded to two decimal places. Notes: ^a^ These values represent the number of non-susceptible isolates and the proportion of all isolates that were non-susceptible to lefamulin (showing resistance and intermediate resistance to lefamulin). Abbreviations: MIC, minimum inhibitory concentration; MIC_50_, MIC for inhibiting 50% of the isolates; MIC_90_, MIC for inhibiting 90% of the isolates; MDR, multi-drug resistant; mg/L, milligram/liter; MSSA, methicillin-susceptible *S. aureus*; MRSA, methicillin-resistant *S. aureus*; N, number of isolates tested; *n*, number of resistant isolates; NA, not available; R, resistant; *S. aureus*, *Staphylococcus aureus*.

**Table 4:** Resistance of *M. catarrhalis* isolates to lefamulin.

Author *	Year	Isolates	N	MIC Range/Value (mg/L)	MIC_50_ (mg/L)	MIC_90_ (mg/L)	Resistance *n* (% **)
Ding [46]	2025	*M. catarrhalis*	81	≤0.015–0.5	0.125	0.5	0 (0) ^a^
Paukner [42]	2023	*M. catarrhalis*	1958	0.008–0.5	0.06	0.12	NA
		β-lactamase positive	1490	0.008–0.5	0.06	0.12	NA
Taylor [38]	2021	*M. catarrhalis*	95	≤0.015–0.12	0.06	0.12	NA
Wu [36]	2020	*M. catarrhalis*	54	≤0.015–0.5	0.25	0.25	NA
Paukner [41]	2013	*M. catarrhalis*	253	≤0.008–0.5	0.12	0.25	NA
Sader [40]	2011	*M. catarrhalis*	50	0.015–0.12	0.06	0.12	NA

* Studies are presented in descending chronological order (and alphabetical order within a year). ** All percentage figures were rounded to one decimal place, except the values <0.1 that were rounded to two decimal places. ^a^ The resistance breakpoint of MIC > 0.5 mg/L was used. Abbreviations: *M. catarrhalis*, *Moraxella catarrhalis*; MIC, minimum inhibitory concentration; MIC_50_, concentration inhibiting 50% of isolates; MIC_90_, concentration inhibiting 90% of isolates; mg/L, milligram/liter; N, number of isolates tested; *n*, number of resistant isolates; NA, not available.

**Table 5:** Resistance of *Streptococcus* spp. isolates (except *S. pneumoniae*) to lefamulin.

Author *	Year	Isolates	N	MIC Range/Value (mg/L)	MIC_50_ (mg/L)	MIC_90_ (mg/L)	Resistance *n* (% **)
Paukner [42]	2023	β-hemolytic *Streptococcus* spp.	128	0.015–0.25	0.03	0.06	NA
		*S. agalactiae*	72	0.015–0.12	0.03	0.06	NA
		*S. pyogenes*	39	0.015–0.25	0.03	0.03	NA
		*S. dysgalactiae*	17	0.03–0.12	0.06	0.06	NA
		Viridans group *Streptococcus* spp. ^a^	50	0.008–1	0.06	0.25	NA
		*S. mitis* group	23	0.015–1	0.12	0.5	NA
		*S. anginosus* group	22	0.008–0.5	0.06	0.25	NA
		*S. salivarius* group	5	0.015–0.12	0.06	NA	NA
Wu [36]	2020	*S. pyogenes*	30	≤0.015	≤0.015	≤0.015	NA
		*S. agalactiae*	13	≤0.015–0.03	≤0.015	0.03	NA
Mendes [44]	2019	*S. agalactiae*	168	≤0.008–16	0.03	0.03	NA
		*S. pyogenes*	165	≤0.008–0.03	0.015	0.03	NA
		*S. dysgalactiae*	56	0.015–0.06	0.03	0.06	NA
		*S. mitis* group	48	0.015–0.5	0.12	0.5	NA
		*S. anginosus* group	44	≤0.008–0.5	0.06	0.25	NA
		*S. salivarius/S. vestibularis* group	40	≤0.008–0.25	0.06	0.12	NA
		*S. gallolyticus*	34	0.06–>32	1	2	NA
		*S. lutetiensis*	7	≤0.008–1	0.015	1	NA
		*S. bovis*	3	≤0.008–0.25	≤0.008	0.25	NA
		*S. equinus*	2	0.015	0.015	0.015	NA
Paukner [41]	2013	β-hemolytic *Streptococcus* spp.	763	≤0.008–16	0.03	0.03	NA
		*S. agalactiae*	334	≤0.008–16	0.03	0.03	NA
		*S. pyogenes*	267	≤0.008–0.06	0.03	0.03	NA
		Viridans group *Streptococcus* spp.	245	≤0.008–2	0.12	0.5	NA
Sader [39]	2012	β-hemolytic *Streptococcus* spp.	354	≤0.008–0.5	0.03	0.06	NA
		group A streptococci	155	0.015–0.12	0.03	0.03	NA
		group B streptococci	117	≤0.008–0.12	0.03	0.06	NA
		other β-hemolytic *Streptococci* spp.	82	0.015–0.5	0.03	0.06	NA
		Viridans group *Streptococcus* spp.	120	≤0.008–4	0.12	0.5	NA

* Studies are presented in descending chronological order (and alphabetical order within a year). ** All percentage figures were rounded to one decimal place, except the values <0.1 that were rounded to two decimal places. Notes: ^a^ excluding *S. bovis group.* Abbreviations: MIC, minimum inhibitory concentration; MIC_50_, concentration inhibiting 50% of isolates; MIC_90_, concentration inhibiting 90% of isolates; mg/L, milligram/liter; N, number of isolates tested; *n*, number of resistant isolates; NA, not available; *S. agalactiae*, *Streptococcus agalactiae*; *S. anginosus*, *Streptococcus anginosus*; *S. bovis*, *Streptococcus bovis*; *S. dysgalactiae*, *Streptococcus dysgalactiae*; *S. equinus*, *Streptococcus equinus*; *S. gallolyticus*, *Streptococcus gallolyticus*; *S. lutetiensis*, *Streptococcus lutetiensis*; *S. mitis*, *Streptococcus mitis*; *S. pyogenes*, *Streptococcus pyogenes*; *S. salivarius*, *Streptococcus salivarius*; *S. vestibularis*, *Streptococcus vestibularis*; spp., species.

**Table 6:** Resistance of other isolates to lefamulin.

Author *	Year	Isolates	N	MIC Range/Value (mg/L)	MIC_50_ (mg/L)	MIC_90_ (mg/L)	Resistance *n* (% **)
**Atypical**							
Ding [46]	2025	*M. pneumoniae*	15	≤0.03	≤0.03	≤0.03	NA
Wu [36]	2020	*M. pneumoniae*	54	≤0.015–0.03	0.03	0.03	NA
Waites [37]	2016	*M. pneumoniae*	60	≤0.001–0.008	≤0.001	0.002	NA
		marcolide-S	18	≤0.001	≤0.001	≤0.001	NA
		marcolide-R	42	≤0.001–0.008	0.002	0.002	NA
Sader [40]	2011	*M. pneumoniae*	50	≤0.003–0.024	0.006	0.006	NA
		*C. pneumoniae*	50	0.02–0.08	0.02	0.04	NA
		*L. pneumophila*	30	0.06–1	0.06	0.5	NA
***Haemophilus* spp. (except *H. influenzae*)**							
Paukner [42]	2023	*H. parainfluenzae*	310	0.12–≥8	1	4	NA
Wu [36]	2020	*H. parainfluenzae*	10	0.015–2	0.5	1	NA
***Staphylococcus* spp. (except *S. aureus*)**							
Wu [36]	2020	*S. epidermidis*, methicillin-R	15	≤0.015–0.125	≤0.015	0.06	NA
		*S. epidermidis*, methicillin-S	15	≤0.015–0.06	≤0.015	0.03	NA
Paukner [41]	2013	coagulase-negative *Staphylococci*	878	≤0.008–>16	0.06	0.12	NA
		methicillin-R	637	≤0.008–>16	0.06	0.12	NA
		methicillin-S	241	≤0.008–4	0.06	0.06	NA
Sader [39]	2012	coagulase-negative *Staphylococci*	204	0.015–>16	0.06	0.12	NA
		methicillin-resistant	104	0.015–>16	0.06	0.25	NA
		methicillin-susceptible	100	0.015–8	0.06	0.12	NA
***Enterococcus* spp.**							
Paukner [41]	2013	*E. faecium*	536	0.015–>16	0.12	4	NA
		vancomycin-NS	304	0.015–>16	0.06	0.25	NA
		vancomycin-S	232	0.03–>16	0.12	>16	NA
Sader [39]	2012	*E. faecium*	214	0.015–>16	0.12	16	NA
		vancomycin-S	129	0.03–>16	0.12	16	NA
		vancomycin-NS	85	0.015–>16	0.12	2	NA
		*E. faecalis*	50	4–>16	>16	>16	NA
		*Enterococcus* spp.	22	≤0.008–>16	4	>16	NA

* Studies are presented in descending chronological order (and alphabetical order within a year). ** All percentage figures were rounded to one decimal place, except the values <0.1 that were rounded to two decimal places. Abbreviations: *C. pneumoniae*, *Chlamydia pneumoniae*; *E. faecalis*, *Enterococcus faecalis*; *E. faecium*, *Enterococcus faecium*; *Enterococcus* spp., *Enterococcus* species; *H. parainfluenzae*, *Haemophilus parainfluenzae*; *L. pneumophila*, *Legionella pneumophila*; *M. catarrhalis*, *Moraxella catarrhalis*; *M. pneumoniae*, *Mycoplasma pneumoniae*; MIC, minimum inhibitory concentration; MIC_50_, concentration inhibiting 50% of isolates; MIC_90_, concentration inhibiting 90% of isolates; mg/L, milligram/liter; N, number of isolates tested; *n*, number of resistant isolates; NA, not available; NS, non-susceptible; R, resistant; S, susceptible; *S. epidermidis*, *Staphylococcus epidermidis*; spp., species.

**Table 7:** Comparative resistance or non-susceptibility of CABP-causing isolates to selected antimicrobials.

Author *	Year	Isolates	N	Resistance, % **^,^***						
				Lefamulin	Penicillin ****	Amoxicillin-Clavulanate	Ceftriaxone	Azithromycin	Levofloxacin	Moxifloxacin
Ding [46]	2025	*S. pneumoniae*	529	0	54.3, I: 20.4	–	–	94.5, I: 2.1	1.7, I: 0.4	0.8, I: 0.9
		penicillin-R ^a^	287	0	100	–	–	99.3, I: 0.4	1.7	0.3, I: 1.4
		penicillin-S ^b^	134	0	0	–	–	82.1, I: 2.1	1.5, I: 0.7	0.7, I: 0.8
		penicillin-I ^c^	108	0	I: 100			97.2, I: 0.9	1.9, I: 0.9	1.9
		*H. influenzae*	121	0	–	–	0	NS: 34.4	0	0
		β-lactamase positive	63	0	–	–	0	NS: 66.7	0	0
		β-lactamase negative	58	0	–	–	0	NS: 3.4	0	0
		*S. aureus*	306	NS: 2.3	–	–	–	54.9, I: 0.3	17	17.3, I: 0.7
		MRSA	118	NS: 3.4	–	–	–	68.6	31.4	32.2, I: 0.9
		MSSA	188	1.6	–	–	–	46.3, I: 0.5	8	8, I: 0.5
		*M. catarrhalis*	81	0	–	–	0	NS: 23.5	0	6.2
		*M. pneumoniae*	15	NA	–	–	–	86.7	0	0
Paukner [42]	2023	*S. pneumoniae* ^d^	10,586	0.09	–	NS: 7	–	NS: 37	–	NS: 1.2
		MDR ^e^	2342	0.2	–	NS: 25.9	–	NS: 99	–	NS: 3.4
		penicillin-R ^a^	1519	0	–	NS: 48.3	–	NS: 84.7	–	NS: 3.5
		XDR ^e^	553	0	–	NS: 84.4	–	NS: 100	–	NS: 8.7
		*H. influenzae*	3496	0.9	–	NS: 9	–	NS: 2.1	–	NS: 0.9
		β-lactamase positive	816	1.3	–	NS: 86.6	–	NS: 4.4	–	NS: 0.6
		*S. aureus*	3056	0.4	–	–	–	NS: 43.3	–	NS: 27
		MRSA	987	0.6	–	–	–	NS: 76.6	–	NS: 69.9
		MDR ^e^	610	0.2	–	–	–	100	–	100
		*M. catarrhalis*	1958	ΝA	–	0	–	NS: 0.2	–	–
		β-lactamase positive	1490	ΝA	–	0	–	NS: 0.3	–	–
		*H. parainfluenzae*	310	ΝA	–	NS: 3.9	–	NS: 6.1	–	NS: 17.1
Taylor [38]	2021	*S. pneumoniae*	482	0	4.4, I: 13.2	0.4, I: 2.1	0	–	–	I: 0.2
		penicillin-S ^b^	397	0	0	0	0	–	–	0
		clarithromycin-R ^f^	110	0	15.5, I: 32.7	1.8, I: 7.3	0	–	–	I: 0.9
		doxycycline-R ^g^	67	0	26.9, I: 49.2	3, I: 11.9	0	–	–	I: 1.5
		penicillin-I ^c^	64	0	I: 100	0	0	–	–	I: 1.6
		penicillin-R ^a^	21	0	100	9.5, I: 47.6	0	–	–	0
		*H. influenzae*	99	1	–	1	0	–	–	0
		β-lactamase positive ^h^	69	1.4	–	0	0	–	–	0
		β-lactamase negative	30	0	–	3.3	0	–	–	0
		*M. catarrhalis*	95	ΝA	–	0	0	–	–	–
		MSSA	70	4.3	–	–	–	–	–	8.6
		MRSA	130	ΝA	–	–	–	–	–	77.7, I: 3.1
Wu [36]	2020	*S. pneumoniae* penicillin-R ^a^	118	0	100	–	45.8, I: 6.7	100	1.7	0.8, I: 0.9
		*S. pneumoniae* penicillin-I ^e^	40	0	I: 100	–	0	100	0	0 0
		*S. pneumoniae* penicillin-S ^b^	40	0	0	–	0	97.5	3.3, I: 0.2	I: 2.5
		*H. influenzae* β-lactamase negative	48	0	–	–	0	NS: 2.1	0	0
		*H. influenzae* β-lactamase positive	48	0	–	–	0	NS: 72.9	0	0
		MRSA	60	NA	–	–	–	85	53.3, I: 1.7	48.3, I: 3.4
		MSSA	61	0	–	–	–	55.7	3.3	3.3
		*M. catarrhalis*	54	NA	–	–	0	NS: 33.3	0	–
		*M. pneumoniae*	54	NA	–	–	–	94.4	–	0
		*H. parainfluenzae*	10	NA	–	–	0	NS: 10	NS: 20	NS: 40
Paukner [43]	2019	*S. pneumoniae*	3923	0.1	12, I: 22.4	3.5, I: 2.9 ^i^	0.9, I: 3	33.6, I: 0.7	1.1, I: 0.1	0.6, I: 0.5 ^i^
		MDR ^e^	821	0.2	42.3, I: 41.4	14.1, I: 11.7	4.4, I: 14	97.7, I: 1.1	3.3, I: 0.2	1.2, I: 1.6 ^i^
		*S. aureus*	2919	0.4	–	–	–	39.4, I: 1.4	26.9, I: 0.9	19.1, I: 7.5 ^i^
		MRSA	938	0.6	–	–	–	75.5, I: 0.7	72.3, I: 1.7	51.9, I: 18.5 ^i^
		*H. influenzae*	1086	0.9	–	2	0	NS: 1.2	NS: 0.4	NS: 0.4 ^i^
		*M. catarrhalis*	667	NA	–	0	0	0	0	0.5 ^i, j^
Mendes [45]	2016	*S. pneumoniae*	822	0.4	0.5, I: 10.3 ^k^	–	1.6, I: 5.8	–	1.1	–
Paukner [41]	2013	*S. pneumoniae*	1473	0.2	21.2, I: 17.5	–	1.2, I: 7.5	36.6, I: 0.8	1	0
		*H. influenzae*	360	1.7	–	–	0	NS: 1.7	–	0
		*M. catarrhalis*	253	NA	–	–	0	NS: 0.4	–	0 ^l^
		*S. aureus*	2527	0.5	–	–	–	–	35.3, I: 1.3	–
		MRSA	2370	0.8	–	–	–	–	71.2, I: 2	–
Sader [40]	2011	*S. pneumoniae* ^d^	157	0	33.1, I: 22.5	–	–	50.3	1.3, I: 0.6	–
		penicillin-S ^b^	54	0	0	–	–	16.7	I: 1.9	–
		penicillin-R ^a^	52	0	100	–	–	75	1.9	–
		penicillin-I ^c^	51	0	I: 100	–	–	60.8	2	–
		*H. influenzae*	102	0	–	0	–	0	0	–
		β-lactamase negative	51	0	–	0	–	0	0	–
		β-lactamase positive	51	0	–	0	–	0	0	–
		*M. catarrhalis*	50	NA	–	0 ^l^	–	0	0	–

* Studies are presented in descending chronological order. ** All percentage figures were rounded to one decimal place, except the values < 0.1 that were rounded to two decimal places. *** Susceptibility data for lefamulin were based on CLSI and EUCAST criteria, as described in Section 2 [48,49]. Susceptibility data for penicillin, amoxicillin-clavulanate, ceftriaxone, azithromycin, levofloxacin, and moxifloxacin were based on CLSI criteria in effect at the time of the original studies, unless noted otherwise. **** Breakpoints for oral penicillin were applied, unless noted otherwise. Notes: ^a^ MIC ≥ 2 mg/L, ^b^ MIC ≤ 0.06 mg/L, ^c^ MIC ≤ 0.12–1 mg/L, ^d^ oral, non-meningitis, ^e^ MDR and XDR status was based on nonsusceptibility to ≥3 and ≥5 classes, respectively, of the following antimicrobial agents, as described by Golden et al. and applying the following breakpoints: penicillin (MIC ≥ 4 mg/L), ceftriaxone (MIC ≥ 2 mg/L), erythromycin (MIC ≥ 0.5 mg/L), clindamycin (MIC ≥ 0.5 mg/L), levofloxacin (MIC ≥ 4 mg/L), tetracycline (MIC ≥ 2 mg/L), and trimethoprim-sulfamethoxazole (MIC ≥ 1 mg/L), ^f^ clarithromycin resistant as MIC ≥ 1 mg/L, ^g^ doxycycline -resistant as MIC ≥ 1 mg/L, ^h^ β-lactamase production for *H. influenzae* was analyzed according to the 2016 Clinical Microbiology Procedures Handbook, ^i^ A subset of the total isolates (N) was tested against this antimicrobial agent (not the whole population of isolates), ^j^ Based on EUCAST 2018 criteria, as applied by the authors in the original study [50], ^k^ Breakpoints for parenteral penicillin were applied (i.e., susceptible at 2 g/mL, intermediate at 4 g/mL, and resistant at 8 g/mL), ^l^ Based on EUCAST 2011 criteria, as applied by the authors in the original study [51]. Abbreviations: R, resistant; S, susceptible; I, Intermediate; NS, non-susceptible; NA; not applicable (there are no MIC breakpoints available for this antimicrobial agent or resistance percentages could not be calculated using the data available in the original study); *S. pneumoniae*, *Streptococcus pneumoniae*; *H. influenzae*, *Haemophilus influenzae*; *M. catarrhalis*, *Moraxella catarrhalis*; *S. aureus*, *Staphylococcus aureus*; MSSA, methicillin-sensitive *Staphylococcus aureus*; MRSA, methicillin-resistant *Staphylococcus aureus*; *H. parainfluenzae*, *Haemophilus parainfluenzae*; *M. pneumoniae*, *Mycoplasma pneumoniae*; MDR, multi-drug resistant; XDR, extensively drug resistant.

## Data Availability

No new data were created or analyzed in this study.

## Supplementary Materials

The following supporting information can be downloaded at: https://www.mdpi.com/article/10.3390/antibiotics15010058/s1, Supplementary File S1: Detailed search strategies used in each resource as of 14 October 2025.

## Abbreviations

AMR	antimicrobial resistance
CABP	community-acquired bacterial pneumonia
CAP	community-acquired pneumonia
CLSI	Clinical and Laboratory Standards Institute
DOIs	digital object identifiers
ECR	early clinical response
EMA	European Medicines Agency
EUCAST	European Committee on Antimicrobial Susceptibility Testing
FDA	US Food and Drug Administration
GI	gastrointestinal
IACR	investigator’s assessment of clinical response
IV	intravenous
MDR	multidrug-resistant
MIC	minimum inhibitory concentration
MIC_50_	minimum inhibitory concentration required to inhibit 50% of organisms
MIC_90_	minimum inhibitory concentration required to inhibit 90% of organisms
MRSA	methicillin-resistant S. aureus
MSSA	methicillin-susceptible S. aureus
PO	oral
PTC	peptidyl transferase center
XDR	extensively drug-resistant
