# Supplementary material for: Resistance to Lefamulin: An Evaluation of Data from In Vitro Antimicrobial Susceptibility Studies
Source: Antibiotics (Basel). 2026 Jan 5;15(1):58. doi: 10.3390/antibiotics15010058 (PMC12838286; doi:10.3390/antibiotics15010058)
Supplement: Supplementary file 1 [file antibiotics-15-00058-s001.zip › antibiotics-4000006-supplementary.pdf]

Supplementary file S1. Detailed search strategies used in each resource as of 14 October 2025

Resource	Search string	Results
Embase	('lefamulin'/exp OR 'lefamulin' OR 'bc-3781'/exp OR 'bc-3781') AND ('antibiotic resistance'/exp OR 'antibiotic resistance' OR 'resistance'/exp OR resistance OR 'non-susceptibility' OR 'non susceptibility' OR nonsusceptibility OR 'reduced susceptibility') AND ('mic'/exp OR mic OR 'minimum inhibitory concentration'/exp OR 'minimum inhibitory concentration' OR 'disc diffusion'/exp OR 'disc diffusion')	81
PubMed	("lefamulin" OR "BC-3781") AND ("antibiotic resistance" OR resistance OR "non-susceptibility" OR "non susceptibility" OR nonsusceptibility OR "reduced susceptibility") AND (MIC OR "minimum inhibitory concentration" OR "disc diffusion")	26
PubMed central	("lefamulin"[tiab] OR "bc-3781"[tiab]) AND ("antibiotic resistance"[tiab] OR resistance[tiab] OR "non-susceptibility"[tiab] OR "reduced susceptibility"[tiab]) AND ("mic"[tiab] OR "minimum inhibitory concentration"[tiab] OR "disc diffusion"[tiab])	21
Scopus	("lefamulin" OR "BC-3781") AND ("antibiotic resistance" OR resistance OR "non-susceptibility" OR "non susceptibility" OR nonsusceptibility OR "reduced susceptibility") AND (MIC OR "minimum inhibitory concentration" OR "disc diffusion")	64
Web of Science	("lefamulin" OR "BC-3781") AND ("antibiotic resistance" OR resistance OR "non-susceptibility" OR "non susceptibility" OR newsusceptibility OR "reduced susceptibility") AND (MIC OR "minimum inhibitory concentration" OR "disc diffusion")	32
